# Is Fragmented Cancer Care Associated With Medical Expenditure? Nationwide Evidence From Patients With Lung Cancer Using National Insurance Claim Data

**DOI:** 10.3389/ijph.2023.1606000

**Published:** 2023-07-06

**Authors:** Kyu-Tae Han, Sun Jung Kim

**Affiliations:** ^1^ Division of Cancer Control and Policy, National Cancer Control Institute, National Cancer Center, Goyang, Republic of Korea; ^2^ Department of Health Administration and Management, College of Medical Science, Soonchunhyang University, Asan, Republic of Korea; ^3^ Center for Healthcare Management Science, Soonchunhyang University, Asan, Republic of Korea; ^4^ Department of Software Convergence, Soonchunhyang University, Asan, Republic of Korea

**Keywords:** moral hazard, cancer care, fragmented care, medical expenditures, health service research

## Abstract

**Objectives:** We aimed to investigate the association between fragmented cancer care in the early phase after cancer diagnosis and patient outcomes using national insurance claim data.

**Methods:** We identified National Health Insurance beneficiaries diagnosed with lung cancer in South Korea from 2010 to 2014. We included 1,364 lung cancer patients with reduced immortal time bias and heterogeneity. We performed multiple regression analysis using a generalized estimate equation with a gamma distribution for medical expenditures.

**Results:** Among the 1,364 patients with lung cancer, 12.8% had fragmented cancer care. Healthcare costs were higher in fragmented cancer care for both during diagnosis to 365 days and diagnosis to 1,825 days. Linear regression results showed that fragmented cancer care was associated with 1.162 times higher costs during the period from diagnosis to 365 days and 1.163 times the cost for the period from diagnosis to 1,825 days.

**Conclusion:** We found fragmented cancer care is associated with higher medical expenditure. Future health policy should consider the limitation of patients’ free will when opting for fragmented cancer care, as there are currently no control mechanisms.

## Introduction

Cancer is a significant cause of death worldwide and is a predominant modern public health concern [1]. Over the past decades, cancer has been the leading cause of death in South Korea. In 2019, there were 29,960 lung cancer cases (out of 254,718 cancer cases; 67.9% were men) and 18,574 deaths (out of 81,203 deaths; 73.7% were men) in South Korea [2]. Age-standardized lung cancer mortality rate was 15.6 per 100,000 individuals in 2019, making it the leading cause of cancer-related deaths [2]. Both men and women aged 60 years or above have been projected to have the highest mortality rates from lung cancer [3]. As South Korea experiences a rapidly aging society, cancer incidence and deaths will increase, necessitating the systematic management of cancers and identifying the factors associated with patient outcomes [4, 5] and overall medical expenditures [6, 7].

Fragmentation of cancer care (FC) is receiving cancer care for a disease process at more than one institution. The Institute of Medicine’s report emphasized FC as a critical problem in the cancer care system and a priority area for patient-centered initiatives [8]. FC is reportedly associated with delays in receiving cancer treatment, higher readmission rates, and worse overall survival in complex oncological procedures [9–12].

In South Korea, recent studies have found that FC was associated with higher mortality among patients with various cancers [13–15]. Moreover, efforts to decrease FC and integrate complex cancer care within appropriate healthcare delivery systems may improve survivorship among patients with cancer [13, 14]. In South Korea, there is no strict gatekeeping system for controlling healthcare utilization, and it is relatively easy for patients to access primary and secondary care and services in tertiary hospitals [16]. Patient demand is concentrated in high-volume tertiary hospitals in the capital area [ [17–19], where they can receive multidisciplinary therapy and centralized cancer care. These have been emphasized by the National Comprehensive Cancer Network guidelines and are primarily performed at these hospitals [20, 21]. Furthermore, after initial treatment, medical staff may recommend a transfer, or the treated patient may relocate to a hospital for better treatment conditions [22, 23].

Lack of coordinated cancer care between hospitals may cause delays in initiating treatment and are likely to lead to fragmented cancer care because healthcare services cannot be appropriately accessed [24, 25]. This lack of a delivery mechanism for limiting patients’ free will to use FC and the absence of a gatekeeping system have been highlighted as causes of quality issues and inefficiency in healthcare delivery [26]. As mentioned above, FC is associated with a high probability of having multiple caregivers, which may lead to lapses in quality and safety [27]. FC introduces a high rate of complications, particularly for older adults with chronic health conditions, which can result in adverse events and high healthcare costs [28–30].

While FC has been studied in various cancers regarding its clinical outcomes, there is a lack of research focused on understanding how FC is associated with medical expenditure. To the authors’ knowledge, no study has examined the association between FC and medical expenditure. To address this research gap, this study aimed to investigate whether FC is associated with medical expenditure among patients with lung cancer in South Korea using a representative sample cohort dataset.

## Methods

### Study Population

The data used in this study was the National Health Insurance sampled cohort 2.0, collected via random sampling for people stratified according to sex, age, region, type of insurance, and insurance premium in 2006. These data included general characteristics and medical records of patients tracked between 2002 and 2019 (*N* = 1,000,000). Among these sample cohorts, we included patients according to the following criteria.

**FIGURE 1:**
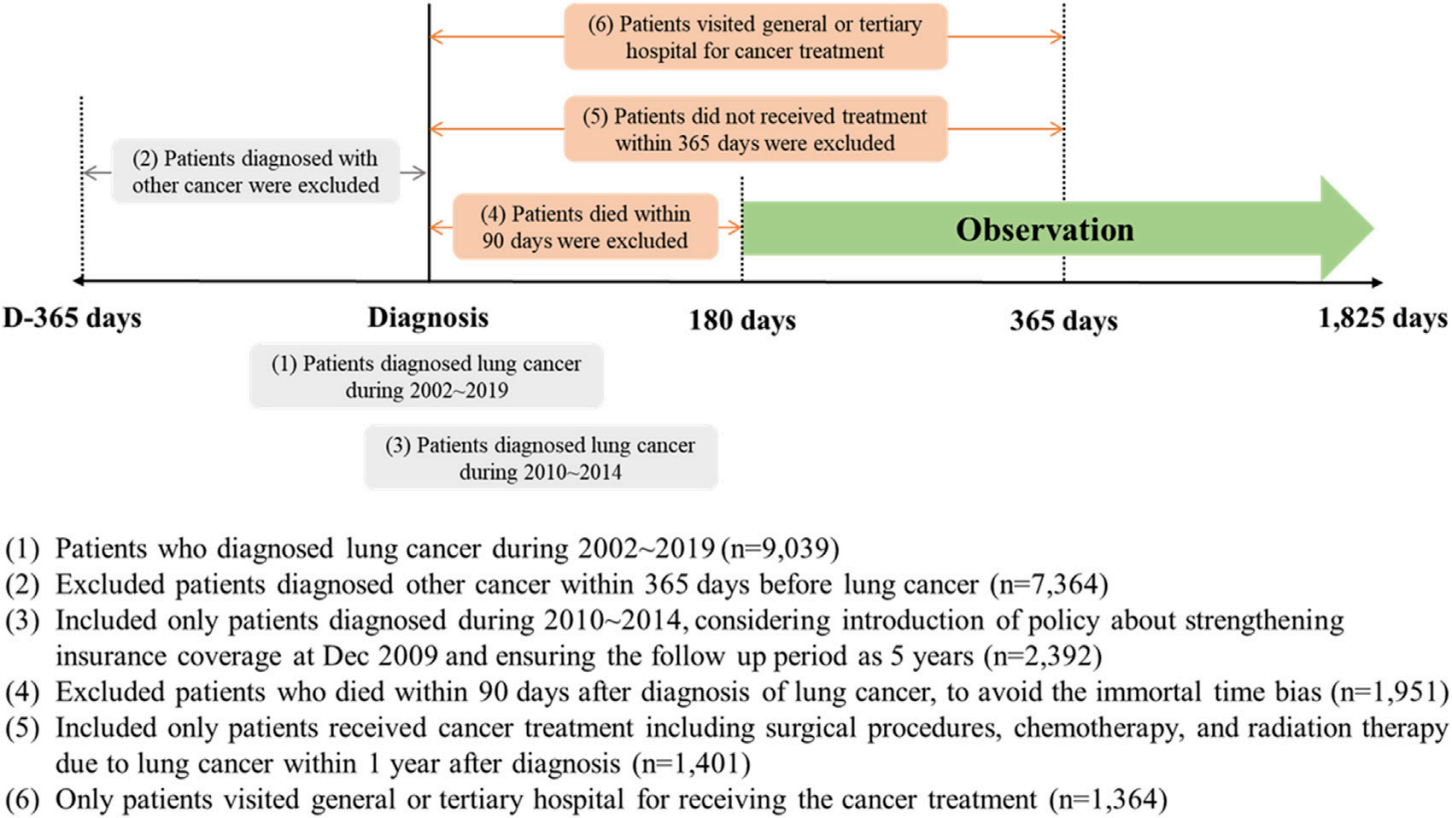
The flow of the selection of the study population to investigate the impact of fragmented cancer care on short- or long-term medical expenditures in patients with lung cancer. (National Health Insurance sampled cohort, South Korea. 2002–2019).

First, we included newly diagnosed patients with lung cancer based on diagnostic codes (International Classification of Diseases-10: C34) from 2002 to 2019 (*N* = 9,039) and excluded those diagnosed with other cancers within 1 year before lung cancer (*N* = 7,364). Second, policies to strengthen cancer coverage in Korea were gradually introduced between 2004 and 2009, which could affect patient spending. Thus, we only included patients diagnosed from 2010 to 2014 to avoid policy effects and to ensure that the observation period was 5 years after the first diagnosis (*N* = 2,392). Third, we considered patients who did not die within 90 days after the first diagnosis to avoid immortal time bias (*N* = 1,951) and only included those who received cancer treatments such as surgical procedures, chemotherapy, or radiation therapy within 1 year after diagnosis (*N* = 1,401). Finally, we included patients who visited a general or tertiary hospital to receive the first cancer treatment to ensure homogeneity between patients (*N* = 1,364; Figure 1).

### Variables

The dependent variable was medical expenditure according to cancer care. In this study, the patient’s expenditures due to cancer care were categorized into two types by the period after diagnosis (diagnosis to 365 days and diagnosis to 1,825 days); we aimed to compare the impact of FC on medical expenditures on short- or long-term perspectives. Due to the nature of cancer care, all patients could not be observed in the same period after diagnosis due to death, and the cancer care expenditure was converted based on the observation time.

$$Cost,short-term\;\left[Diagnosis\;to\;365\;days\right]=sum\;of\;cancer\;care\;cost\;/\;sum\;of\;the\;observation\;period\;\left(during\;diagnosis\;to\;365\;days\right)\;*\;365\;days$$

$$Cost,long-term\;\left[Diagnosis\;to\;1,825\;days\right]=sum\;of\;cancer\;care\;cost\;/\;sum\;of\;the\;observation\;period\;\left(during\;diagnosis\;to\;1,825\;days\right)\;*\;1,825\;days$$

The variable of interest was FC. To investigate the association between medical expenditures and cancer care and FC, we first identified the medical records of patients who underwent cancer treatment, such as surgical procedures, chemotherapy, or radiation therapy, and identified the medical institution. Then, if patients were provided cancer treatment at multiple hospitals for 1 year after the cancer diagnosis, we defined them as having FC.

Other independent variables were as follows: type or location of the hospital with first cancer treatment, sex, age, types of insurance coverage, economic status, residence area, year of diagnosis, Charlson comorbidity index (CCI), types of treatment, and 5 years mortality. The types and locations of hospitals that received the first cancer treatment were classified into “tertiary hospital” and “general hospital”; or “capital area,” “metropolitan,” and “rural.” The ages were divided into ∼49, 50–59, 60–69, 70–79, and 80∼ years. Approximately 97% of Koreans are covered by National Health Insurance, divided into two categories. First, the National Health Insurance employee group included employees, business owners, and families who pay insurance premiums according to their salaries. The self-employed group included all other individuals who pay insurance premiums according to income, property, and living standards. Second, the remaining 3% were medical-aid clients who were economically or physically vulnerable and did not pay insurance premiums. This reflects the economic level, which was classified into three groups, and the insurance premium was classified into four groups based on distribution. The residence area was classified into “capital area,” “metropolitan,” and “rural.” The CCI is the sum of scores for comorbidity, excluding cancer, in medical records within 365 days after the first cancer diagnosis. The CCI was categorized into three groups: 0–1, 2, and 3∼. The type of treatment was defined according to what treatment the patient received during the observation period. Patients with lung cancer were observed for 5 years based on the initial date of diagnosis, and if they died within 5 years, they were defined as the “Died” group, and those who survived up to 5 years after diagnosis were defined as “Survived.”

### Statistical Analysis

To examine the association between medical expenditures due to cancer care and FC, we first analyzed the general distribution of frequencies and percentages and confirmed the chi-squared test results. Next, we compared medical expenditures’ mean and standard deviation and analysis of variance (ANOVA) to identify the differences according to FC. Finally, we performed multiple regression analysis using a generalized estimate equation (GEE) with a gamma distribution for medical expenditures related to FC with adjusting covariates. In addition, we performed a sensitivity analysis to compare the differences according to the residential area or location of the first cancer treatment. All statistical analyses in this study were performed using SAS statistical software (version 9.4; Cary, NC, United States).

## Results

A total of 1,364 patients with lung cancer were included in the study. Table 1 shows the patient distribution according to fragmented cancer care. Of the patients, 12.8% experienced fragmented cancer care; patients visited more than two hospitals due to cancer treatment after diagnosis (fragmented cancer care, yes = 12.8%, no = 87.2%). Patients who visited tertiary hospitals or hospitals in the capital area for their first cancer treatment received more frequent fragmented care, but it was not statistically significant (*p* = 0.4454; *p* = 0.3227). Patients who lived in rural areas received fragmented cancer care more frequently than patients residing in capital or metropolitan areas (residence area, capital area = 8.7%, metropolitan = 13.5%, rural = 16.3%, *p* = 0.0011). Moreover, patients who received surgical treatment experienced more fragmented cancer care (types of treatment, surgery with chemotherapy or radiation therapy = 21.4%, only surgery = 0.9%, chemotherapy or radiation therapy = 14.3%, *p* < 0.0001). Patients who died within 5 years after a lung cancer diagnosis were likelier to visit multiple hospitals (5 years mortality; died = 16.5%, survived = 5.6%; *p* < 0.0001).

**TABLE 1:** Distribution of fragmented cancer care according to covariates (National Health Insurance sampled cohort, South Korea. 2002–2019).

Variables	Total	Fragmented cancer care				*p*-value
		Yes		No		
		N	%	N	%	
Type of institution for first cancer treatment						
Tertiary hospital	970	128	13.2	842	86.8	0.4454
General hospital	394	46	11.7	348	88.3	
Location of institution for first cancer treatment						
Capital area	834	115	13.8	719	86.2	0.3227
Metropolitan	317	37	11.7	280	88.3	
Rural	213	22	10.3	191	89.7	
Sex						
Male	961	128	13.3	833	86.7	0.3359
Female	403	46	11.4	357	88.6	
Age (Years)						
49 and below	100	12	12.0	88	88.0	0.7681
50–59	250	33	13.2	217	86.8	
60–69	449	64	14.3	385	85.7	
70–79	465	54	11.6	411	88.4	
80 and above	100	11	11.0	89	89.0	
Type of insurance coverage						
Medical-Aid	63	11	17.5	52	82.5	0.4084
NHI, Self-employed	416	56	13.5	360	86.5	
NHI, Employee	885	107	12.1	778	87.9	
Economic status						
Low	320	47	14.7	273	85.3	0.6409
Mid-low	274	35	12.8	239	87.2	
Mid-high	303	38	12.5	265	87.5	
High	467	54	11.6	413	88.4	
Residence area						
Capital area	517	45	8.7	472	91.3	0.0011
Metropolitan	319	43	13.5	276	86.5	
Rural	528	86	16.3	442	83.7	
Year of diagnosis						
2010	250	33	13.2	217	86.8	0.6114
2011	261	30	11.5	231	88.5	
2012	272	42	15.4	230	84.6	
2013	267	33	12.4	234	87.6	
2014	314	36	11.5	278	88.5	
Charlson Comorbidity Index (excluding cancer)						
0–1	410	42	10.2	368	89.8	0.1728
2	326	43	13.2	283	86.8	
3+	628	89	14.2	539	85.8	
Types of treatment						
Surgery with chemotherapy or radiation therapy	294	63	21.4	231	78.6	<0.0001
Only surgery	316	3	0.9	313	99.1	
Chemotherapy or radiation therapy	754	108	14.3	646	85.7	
5 years mortality						
Died	899	148	16.5	751	83.5	<0.0001
Survived	465	26	5.6	439	94.4	
Total	1,364	174	12.8	1,190	87.2	

Table 2 shows the independent variables’ mean and standard deviation of medical expenditures. Patients who received fragmented treatment spent more on cancer care in the short and long term (short-term, yes = 28.86, no = 19.93, *p* = 0.0017; long-term, yes = 139.40, no = 88.71, *p* = 0.0006). The characteristics of the first hospital to receive cancer treatment were unrelated to expenditure, but male or older patients generally spent more on cancer care. Regarding types of treatment, patients with only surgery had low values in medical expenditures on short- or long-term perspectives (short-term, surgery with chemotherapy or radiation therapy = 24.32, only surgery = 9.49, chemotherapy or radiation therapy = 24.66, *p* < 0.0001; long-term, surgery with chemotherapy or radiation therapy = 107.23, only surgery = 60.59, chemotherapy or radiation therapy = 88.14, *p* = 0.0011). Moreover, patients who died within 5 years had more cancer costs than those who survived.

**TABLE 2:** Medical expenditure of cancer care and its comparison by the fragmented cancer care and covariates (National Health Insurance sampled cohort, South Korea. 2002–2019).

Variables	Medical expenditure (million KRW, converted based on observation period)					
	Short-term [Diagnosis to 365 days]		*p*-value	Long-term [Diagnosis ∼ 1,825 days]		*p*-value
	Mean	SD		Mean	SD	
Fragmented care						
Yes	28.86	19.75	0.0017	139.40	104.62	0.0006
No	19.93	17.70		88.71	91.98	
Type of institution for first cancer treatment						
Tertiary hospital	21.30	18.67	0.0287	95.13	97.31	0.0941
General hospital	20.52	17.04		95.30	89.76	
Location of institution for first cancer treatment						
Capital area	20.53	18.75	0.8505	91.57	97.17	0.9686
Metropolitan	20.97	16.88		97.66	89.67	
Rural	23.33	17.86		105.59	94.67	
Sex						
Male	23.10	19.27	<0.0001	104.30	100.70	0.0004
Female	16.22	14.28		73.42	76.22	
Age (Years)						
49 and below	21.90	21.32	0.0192	93.90	104.28	0.0042
50–59	20.24	16.77		91.52	92.20	
60–69	20.96	16.28		92.56	87.86	
70–79	21.61	19.35		98.93	99.29	
80+	20.32	21.10		99.91	105.45	
Type of insurance coverage						
Medical-Aid	23.89	21.90	0.9455	114.39	111.11	0.8772
NHI, Self-employed	22.01	18.39		101.49	94.92	
NHI, Employee	20.43	17.82		90.84	93.82	
Economic status						
Low	22.67	18.23	0.3337	104.28	96.84	0.4962
Mid-low	22.40	19.05		100.04	9654.00	
Mid-high	19.98	17.04		88.95	90.99	
High	19.90	18.35		90.12	95.49	
Residence area						
Capital area	20.95	19.20	0.5647	95.11	99.78	0.3151
Metropolitan	20.14	16.30		89.71	87.93	
Rural	21.75	18.30		98.54	94.75	
Year of diagnosis						
2010	22.74	19.14	0.1311	105.29	102.59	0.0984
2011	22.54	20.31		103.48	104.83	
2012	19.35	17.39		85.51	87.43	
2013	19.50	14.54		87.40	79.53	
2014	21.35	18.95		95.20	98.31	
Charlson Comorbidity Index (excluding cancer)						
0–1	18.36	15.71	0.0163	83.15	86.10	0.1869
2	20.65	17.95		93.70	94.98	
3+	23.05	19.59		103.79	100.01	
Types of treatment						
Surgery with chemotherapy or radiation therapy	24.32	19.43	<0.0001	102.20	107.23	0.0011
Only Surgery	9.49	13.15		27.32	60.59	
Chemotherapy or radiation therapy	24.66	17.54		120.87	88.14	
5 years mortality						
Died	26.09	19.50	<0.0001	132.17	96.92	<0.0001
Survived	11.37	9.75		23.66	25.35	
Total	21.07	18.21		95.18	95.16	

Table 3 shows the multiple regression analysis results using the GEE model. Fragmented cancer care was associated with statistically significant higher short- and long-term cancer care expenditures (short-term, yes = relative risk [RR]: 1.161, 95% confidence intervals [CI]: 1.034–1.306; long-term, yes = RR: 1.163, 95% CI: 1.036–1.306; ref = no). Patients who visited the general hospital for the first treatment had a low risk of short-term costs, but it was not statistically significant in long-term expenditures. Male patients had a high risk of spending, but older patients had a low risk. Patients who had higher CCI scores had a higher risk of spending on short- and long-term perspectives (short-term, 2 = RR: 1.079, 95% CI: 0.974–1.196, 3+ = RR: 1.189, 95% CI: 1.087–1.300; ref = no; long-term, 2 = RR: 1.064, 95% CI: 0.961–1.178, 3+ = RR: 1.159, 95% CI: 1.061–1.266; ref = no). In addition, patients who received chemotherapy or radiation therapy had a higher risk of spending from both perspectives than patients who only underwent surgery, and patients who died within 5 years had a higher risk for expenditures.

**TABLE 3:** Results of multiple regression analysis for short- and long-term medical expenditures due to cancer care (National Health Insurance sampled cohort, South Korea. 2002–2019).

Variables	Medical expenditure (million KRW, converted based on observation period)							
	Short-term [Diagnosis to 365 days]				Long-term [Diagnosis ∼ 1,825 days]			
	RR	LCL	UCL	*p*-value	RR	LCL	UCL	*p*-value
Fragmented cancer care								
Yes	1.162	1.034	1.306	0.0115	1.163	1.036	1.306	0.0104
No	1.000	—	—	—	1.000	—	—	—
Type of institution for first cancer treatment								
Tertiary hospital	1.000	—	—	—	1.000	—	—	—
General hospital	0.896	0.823	0.976	0.0115	0.944	0.868	1.026	0.1731
Location of institution for first cancer treatment								
Capital area	1.000	—	—	—	1.000	—	—	—
Metropolitan	0.968	0.863	1.087	0.5847	1.008	0.897	1.133	0.8920
Rural	0.987	0.872	1.117	0.8405	0.932	0.825	1.054	0.2623
Sex								
Male	1.302	1.198	1.416	<0.0001	1.170	1.077	1.272	0.0002
Female	1.000	—	—	—	1.000	—	—	—
Age (Years)								
49 and below	1.000	—	—	—	1.000	—	—	—
50–59	0.889	0.755	1.048	0.1614	0.970	0.825	1.141	0.7116
60–69	0.854	0.732	0.996	0.0439	0.858	0.737	0.999	0.0485
70–79	0.859	0.735	1.004	0.0567	0.854	0.732	0.997	0.0455
80 and above	0.781	0.639	0.955	0.0161	0.810	0.664	0.988	0.0374
Type of insurance coverage								
Medical-Aid	0.987	0.809	1.204	0.8983	1.073	0.883	1.305	0.4775
NHI, Self-employed	1.024	0.942	1.113	0.5758	1.060	0.976	1.152	0.1680
NHI, Employee	1.000	—	—	—	1.000	—	—	—
Economic status								
Low	1.073	0.961	1.198	0.2106	1.088	0.976	1.213	0.1295
Mid-low	1.081	0.970	1.205	0.1567	1.094	0.984	1.217	0.0979
Mid-high	0.976	0.881	1.083	0.6513	0.995	0.899	1.102	0.9261
High	1.000	—	—	—	1.000	—	—	—
Residence area								
Capital area	1.000	—	—	—	1.000	—	—	—
Metropolitan	0.943	0.834	1.067	0.3514	0.908	0.802	1.028	0.1291
Rural	0.976	0.884	1.079	0.6354	0.951	0.862	1.050	0.3224
Year of diagnosis (per 1 year)	1.004	0.978	1.030	0.7849	0.998	0.973	1.024	0.8916
Charlson Comorbidity Index (excluding cancer)								
0–1	1.000	—	—	—	1.000	—	—	—
2	1.079	0.974	1.196	0.1469	1.064	0.961	1.178	0.2309
3+	1.189	1.087	1.300	0.0001	1.159	1.061	1.266	0.0011
Types of treatment								
Surgery with chemotherapy or radiation therapy	1.869	1.654	2.113	<0.0001	1.976	1.751	2.230	<0.0001
Only Surgery	1.000	—	—	—	1.000	—	—	—
Chemotherapy or radiation therapy	1.669	1.483	1.880	<0.0001	1.918	1.714	2.147	<0.0001
5 years mortality								
Died	1.804	1.627	1.999	<0.0001	4.233	3.837	4.668	<0.0001
Survived	1.000	—	—	—	1.000	—	—	—

**FIGURE 2:**
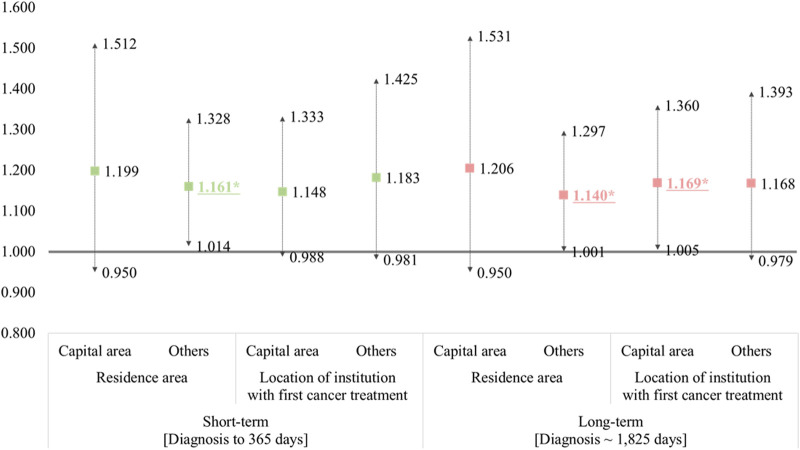
The results of subgroup regression analysis according to the residential area or location of the institution for the first cancer treatment. (National Health Insurance sampled cohort, South Korea. 2002–2019). ^†^ This model was adjusted for other covariates, and the reference group included patients without fragmented care. *Statistically significant results are underlined and asterisked.

Figure 2 shows the multiple regression analysis results based on the location of the patient or hospital that received the initial cancer treatment to compare the association difference between fragmented care and expenditure due to cancer. Regarding each residential area, patients living in non-capital areas (metropolitan or rural areas) showed a significant association between fragmented cancer care and expenditure, but those living in the capital area did not. In addition, concerning the subgroup by hospital location, statistical results were found only in the long-term expenditures of patients who visited hospitals in the capital area.

## Discussion

This study examined the association between fragmented cancer care and medical expenditures among patients with lung cancer. Using a large-scale National Health Insurance sampled cohort dataset, this study found evidence of higher medical expenditure among patients with lung cancer who experienced fragmented cancer care. Furthermore, we observed different patient characteristics associated with the medical expenditures used in the study sample. To our knowledge, no study has yet investigated how fragmented cancer care is associated with medical care. The strength of this study is that it provides new insights and evidence on how fragmented cancer care is associated with medical expenditure, its burden on national healthcare budgets, and plans for efficient healthcare spending and utilization.

The results of our study deliver an essential message to the NHI program and policymakers that fragmented cancer care for patients with lung cancer is associated with worse patient outcomes [14] found in previous research, as well as higher medical expenditures. Under the unique South Korean healthcare delivery system (no limitation on numerous caregivers), healthcare providers must be incentivized to manage effective patient care for those with lung cancer. The patient perspective on care is steadily gaining attention as health systems worldwide aim to deliver high-quality, patient-centered care [31, 32]. However, it should be conducted in a cost-benefit manner. Due to the importance of limited healthcare resource allocation as cancer care continues to expand in an aging society, health policymakers and other stakeholders should be aware of fragmented cancer care groups of patients, and this must be closely monitored. The results of this study merit health policymakers’ attention, and policy concerns should be raised because fragmented cancer care among patients with lung cancer was associated with higher medical expenditure and worse mortality. Patients with lung cancer must be presented with alternatives and well-developed information that optimally guide them through the diagnosis-treatment process and prevent unnecessary fragmented cancer care; currently, there are no mechanisms regulating this issue in South Korea. Salient cancer care dimensions, such as high professional standards, respect, coordination of care, clear and tailored information, rapid diagnosis and treatment, caring caregivers, and so forth, should be emphasized [33]. However, all should be considered financially viable at the national level. Fragmented cancer care has important implications for cancer care coordination and delivery of healthcare, as well as the overall cost of care [34].

Another strength of our study was using a nationwide sample cohort of claims data of patients with lung cancer with a retrospective design that contains all age groups, contributing to the robustness of our study. Interestingly, our study found that the evidence of medical expenditures did not differ among patients with different types of health insurance coverage and economic status, which contradicts other studies [7, 35]. We believe this is because this study only includes health insurance covered services in which health service coverages are sufficiently high for everyone. Future studies should be conducted on non-covered medical expenditure and its association with fragmented cancer care and mortality. Through the results of subgroup analysis, another interesting finding was the differences in fragmented care and its association with expenditure by regional factors. Patients living in non-capital areas were more affected by fragmented care, which is relevant to the concentration of medical resources and patient preferences for cancer treatment. In South Korea, there are many concerns regarding the concentration of medical resources and their use. It is a representative problem posing a moral hazard due to the improvement of coverage and easing burden [18, 36]. We could infer that the influence of fragmented care is more significant in the non-capital area in the short- and long-term, and measures must be considered from the perspective of the distribution and efficiency of medical resources at the national level. In addition, it is necessary to encourage patients to select and use appropriate medical institutions because findings show that the first cancer care institution can affect healthcare expenditure from a long-term perspective. Moreover, an infrastructure must be established for this.

In Korea, cancer patients can visit multiple hospitals to learn more about their clinical status or be provided with superior treatment, regardless of their local healthcare infrastructure. Most of these patients eventually head to a large hospital in the capital area. Under these circumstances of skewness to the capital area, the patient may be able to receive cancer treatment in time, but they could experience problems with continuity of care. From the perspective of large hospitals, patients are forced to be discharged from the perspective of bed rotation for the treatment of other cancer patients, and treatment after the initial treatment will eventually be provided as outpatient care. However, it is not easy for cancer patients whose health is weaker than others to visit the capital area at every treatment.

Unfortunately, these patients lose continuity of care in either treatment or management of their symptoms. Over the past few decades, the South Korean government has perceived the needs and importance of managing the patient`s concentration in the capital area. Many efforts have been made to solve this problem by using regionalization alternatives like the regional cancer center system, but it has not been managed well so far. Thus, based on these findings, optimal alternatives should be considered.

Although this study has several insights and strengths, some limitations are worth noting. First, we used a National Health Insurance sampled cohort dataset in which administrative data, not actual medical records, may have a limited view of fragmented cancer care. Second, the dataset lacks detailed clinical information such as stage or pharmacologic treatments. However, this study contained control variables, including CCI, surgery, radiation, and chemotherapy, which may play a proxy role in patients’ health status. Third, the dataset of this study may not fully capture whether patients received fragmented cancer care based on the patients’ or physicians’ preferences. Fourth, South Korea’s unique insurance and healthcare delivery system may significantly limit the generalizability of the findings to other countries. Furthermore, considering the nature of retrospective data based on claims, the findings presented in this study cannot be used to establish causal associations. Further study should be conducted on how this may affect the fragmented cancer care delivery and its association with mortality in prospective cohort-based settings. Therefore, our results should be interpreted carefully and may not generalize to settings beyond Korea. In addition, we investigated only patients with lung cancer. Therefore, our results will differ from those for other types of cancer, possibly weakening the reliability of our findings. Our findings and previous research showed evidence that fragmented cancer care is associated with worse survival and higher medical expenditure, however, the quality of cancer patients has not been evaluated yet. Further study is required in this matter as well. Finally, the data source of this study does not include cancer patients’ clinical information details, which is one limitation of the administrative dataset. Further study investigating the medical expenditure of fragmented lung cancer care is required using cancer registry data or a cohort dataset controlling for proper severity. Despite these limitations, to our knowledge, this study is the first to analyze and explore fragmented cancer care associated with medical expenditure among patients with lung cancer and provide meaningful results.

### Conclusion

In this study, we found that fragmented cancer care was associated with higher medical expenditure among patients with lung cancer. Future health policy should consider limiting patients’ free care choices when opting for fragmented cancer care, as there are currently no control mechanisms in this regard. Health policymakers should be aware of groups of patients who are at a high risk of expenditures and require monitoring because lung cancer prevalence will continue to increase amid an aging society.
